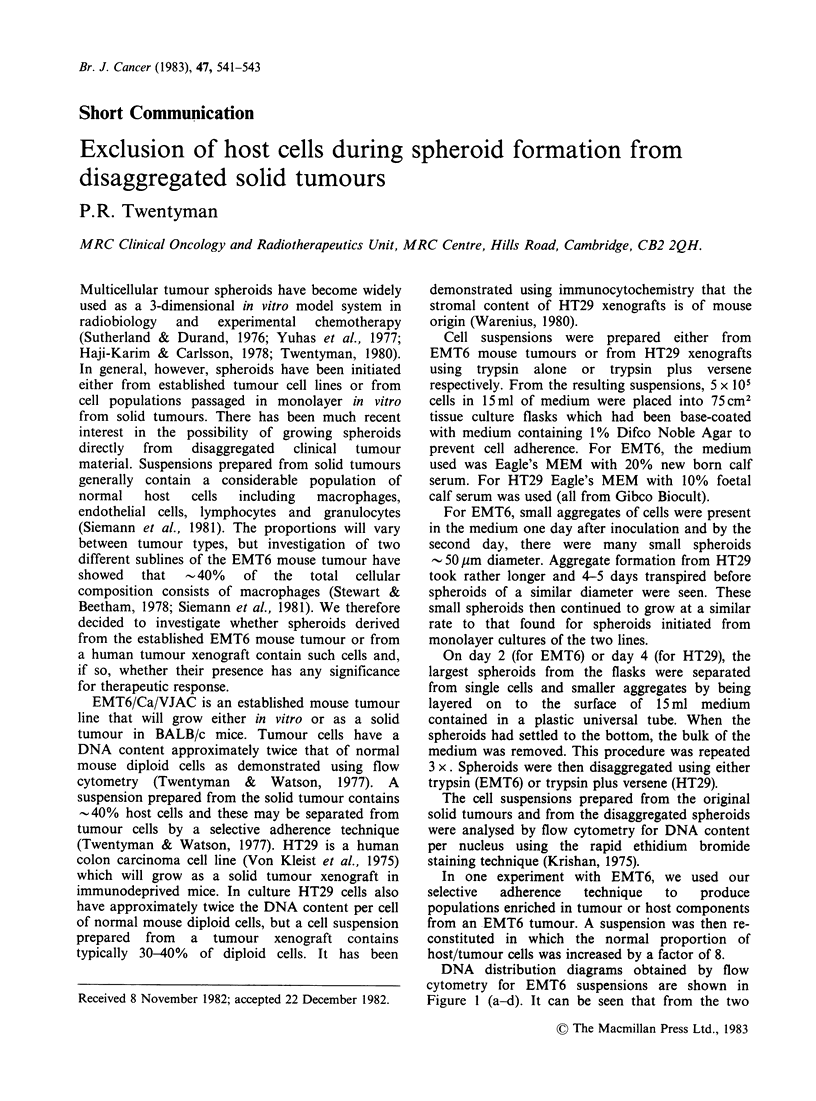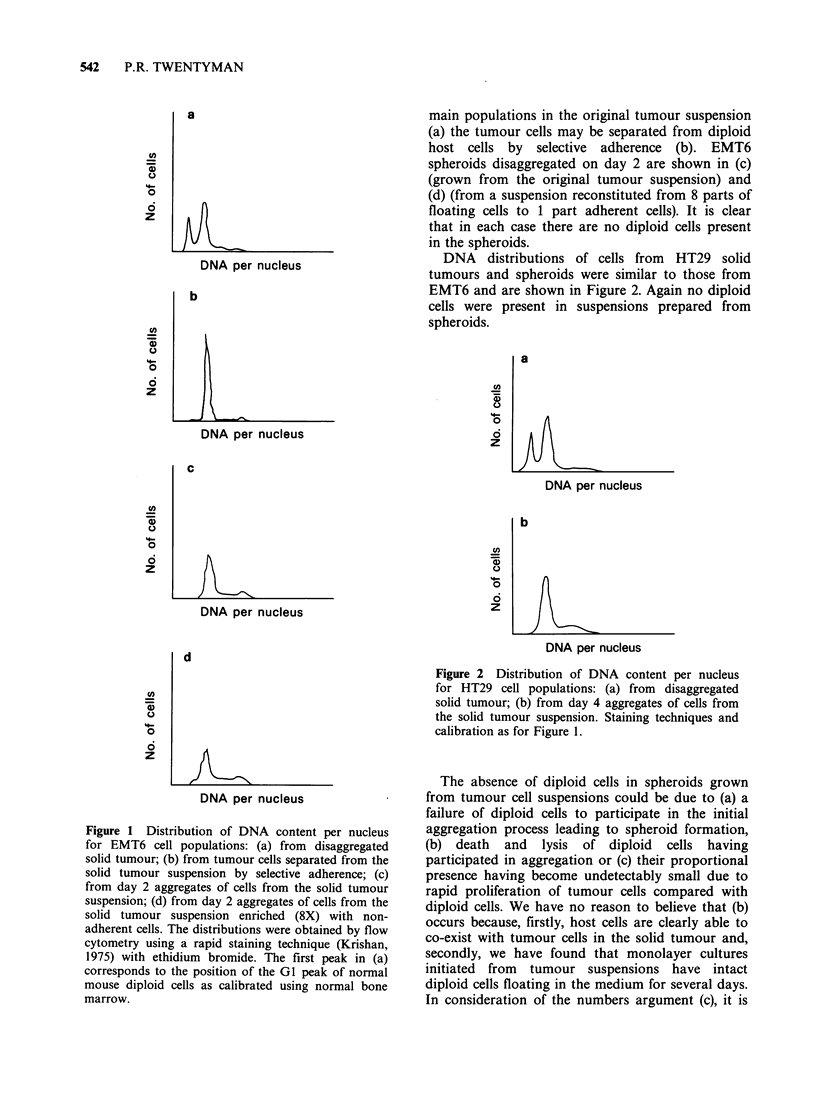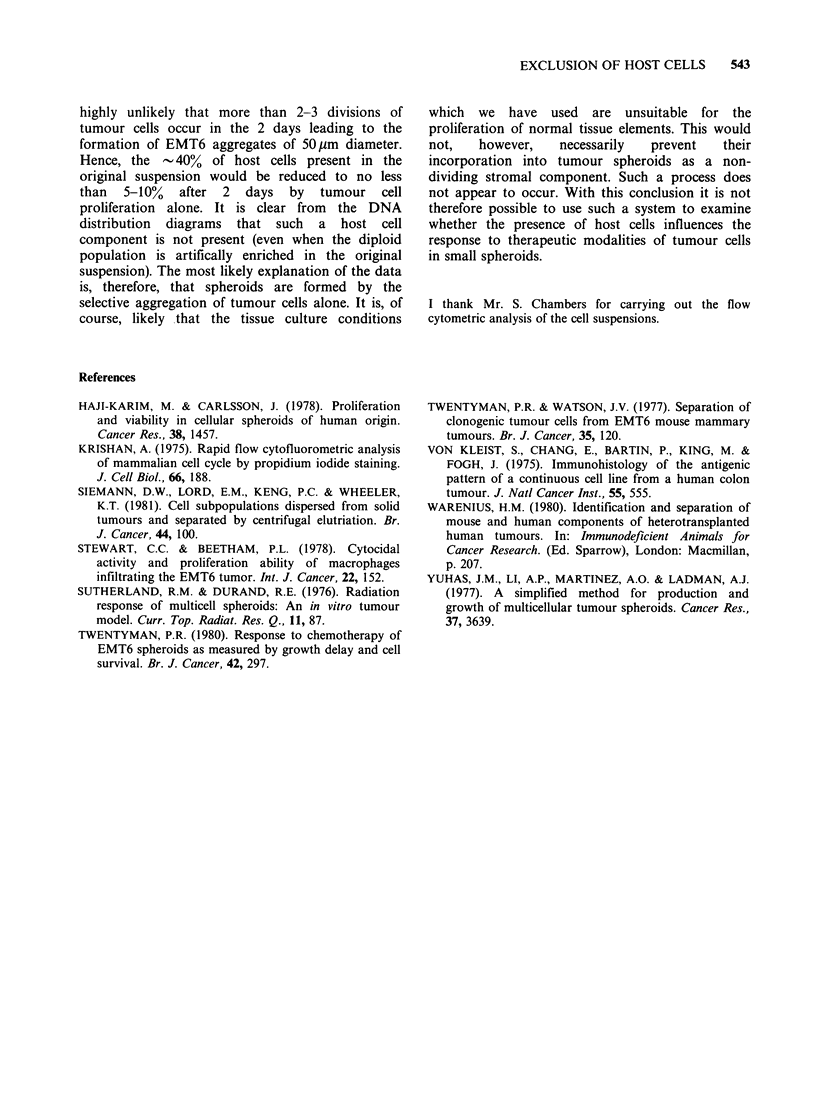# Exclusion of host cells during spheroid formation from disaggregated solid tumours.

**DOI:** 10.1038/bjc.1983.85

**Published:** 1983-04

**Authors:** P. R. Twentyman


*Br. J. Cancer* (1983), **47**, 541–543

### Short Communication

### Exclusion of host cells during spheroid formation from disaggregated solid tumours

P.R. Twentyman

*MRC Clinical Oncology and Radiotherapeutics Unit, MRC Centre, Hills Road, Cambridge, CB2 2QH.*

Multicellular tumour spheroids have become widely used as a 3-dimensional *in vitro* model system in radiobiology and experimental chemotherapy (Sutherland & Durand, 1976; Yuhas *et al.*, 1977; Haji-Karim & Carlsson, 1978; Twentyman, 1980). In general, however, spheroids have been initiated either from established tumour cell lines or from cell populations passaged in monolayer *in vitro* from solid tumours. There has been much recent interest in the possibility of growing spheroids directly from disaggregated clinical tumour material. Suspensions prepared from solid tumours generally contain a considerable population of normal host cells including macrophages, endothelial cells, lymphocytes and granulocytes (Siemann *et al.*, 1981). The proportions will vary between tumour types, but investigation of two different sublines of the EMT6 mouse tumour have showed that ~40% of the total cellular composition consists of macrophages (Stewart & Beetham, 1978; Siemann *et al.*, 1981). We therefore decided to investigate whether spheroids derived from the established EMT6 mouse tumour or from a human tumour xenograft contain such cells and, if so, whether their presence has any significance for therapeutic response.

EMT6/Ca/VJAC is an established mouse tumour line that will grow either *in vitro* or as a solid tumour in BALB/c mice. Tumour cells have a DNA content approximately twice that of normal mouse diploid cells as demonstrated using flow cytometry (Twentyman & Watson, 1977). A suspension prepared from the solid tumour contains ~40% host cells and these may be separated from tumour cells by a selective adherence technique (Twentyman & Watson, 1977). HT29 is a human colon carcinoma cell line (Von Kleist *et al.*, 1975) which will grow as a solid tumour xenograft in immunodeprived mice. In culture HT29 cells also have approximately twice the DNA content per cell of normal mouse diploid cells, but a cell suspension prepared from a tumour xenograft contains typically 30–40% of diploid cells. It has been demonstrated using immunocytochemistry that the stromal content of HT29 xenografts is of mouse origin (Warenius, 1980).

Cell suspensions were prepared either from EMT6 mouse tumours or from HT29 xenografts using trypsin alone or trypsin plus versene respectively. From the resulting suspensions, $5 \times 10^5$ cells in 15 ml of medium were placed into 75 cm$^2$ tissue culture flasks which had been base-coated with medium containing 1% Difco Noble Agar to prevent cell adherence. For EMT6, the medium used was Eagle's MEM with 20% new born calf serum. For HT29 Eagle's MEM with 10% foetal calf serum was used (all from Gibco Biocult).

For EMT6, small aggregates of cells were present in the medium one day after inoculation and by the second day, there were many small spheroids ~50 $\mu$m diameter. Aggregate formation from HT29 took rather longer and 4–5 days transpired before spheroids of a similar diameter were seen. These small spheroids then continued to grow at a similar rate to that found for spheroids initiated from monolayer cultures of the two lines.

On day 2 (for EMT6) or day 4 (for HT29), the largest spheroids from the flasks were separated from single cells and smaller aggregates by being layered on to the surface of 15 ml medium contained in a plastic universal tube. When the spheroids had settled to the bottom, the bulk of the medium was removed. This procedure was repeated 3×. Spheroids were then disaggregated using either trypsin (EMT6) or trypsin plus versene (HT29).

The cell suspensions prepared from the original solid tumours and from the disaggregated spheroids were analysed by flow cytometry for DNA content per nucleus using the rapid ethidium bromide staining technique (Krishan, 1975).

In one experiment with EMT6, we used our selective adherence technique to produce populations enriched in tumour or host components from an EMT6 tumour. A suspension was then re-constituted in which the normal proportion of host/tumour cells was increased by a factor of 8.

DNA distribution diagrams obtained by flow cytometry for EMT6 suspensions are shown in Figure 1 (a–d). It can be seen that from the two

Received 8 November 1982; accepted 22 December 1982.

© The Macmillan Press Ltd., 1983

**Figure 1** Distribution of DNA content per nucleus for EMT6 cell populations: (a) from disaggregated solid tumour; (b) from tumour cells separated from the solid tumour suspension by selective adherence; (c) from day 2 aggregates of cells from the solid tumour suspension; (d) from day 2 aggregates of cells from the solid tumour suspension enriched (8X) with non-adherent cells. The distributions were obtained by flow cytometry using a rapid staining technique (Krishan, 1975) with ethidium bromide. The first peak in (a) corresponds to the position of the G1 peak of normal mouse diploid cells as calibrated using normal bone marrow.

main populations in the original tumour suspension (a) the tumour cells may be separated from diploid host cells by selective adherence (b). EMT6 spheroids disaggregated on day 2 are shown in (c) (grown from the original tumour suspension) and (d) (from a suspension reconstituted from 8 parts of floating cells to 1 part adherent cells). It is clear that in each case there are no diploid cells present in the spheroids.

DNA distributions of cells from HT29 solid tumours and spheroids were similar to those from EMT6 and are shown in Figure 2. Again no diploid cells were present in suspensions prepared from spheroids.

**Figure 2** Distribution of DNA content per nucleus for HT29 cell populations: (a) from disaggregated solid tumour; (b) from day 4 aggregates of cells from the solid tumour suspension. Staining techniques and calibration as for Figure 1.

The absence of diploid cells in spheroids grown from tumour cell suspensions could be due to (a) a failure of diploid cells to participate in the initial aggregation process leading to spheroid formation, (b) death and lysis of diploid cells having participated in aggregation or (c) their proportional presence having become undetectably small due to rapid proliferation of tumour cells compared with diploid cells. We have no reason to believe that (b) occurs because, firstly, host cells are clearly able to co-exist with tumour cells in the solid tumour and, secondly, we have found that monolayer cultures initiated from tumour suspensions have intact diploid cells floating in the medium for several days. In consideration of the numbers argument (c), it is highly unlikely that more than 2–3 divisions of tumour cells occur in the 2 days leading to the formation of EMT6 aggregates of 50 $\mu$m diameter. Hence, the $\sim$40% of host cells present in the original suspension would be reduced to no less than 5–10% after 2 days by tumour cell proliferation alone. It is clear from the DNA distribution diagrams that such a host cell component is not present (even when the diploid population is artifically enriched in the original suspension). The most likely explanation of the data is, therefore, that spheroids are formed by the selective aggregation of tumour cells alone. It is, of course, likely that the tissue culture conditions which we have used are unsuitable for the proliferation of normal tissue elements. This would not, however, necessarily prevent their incorporation into tumour spheroids as a non-dividing stromal component. Such a process does not appear to occur. With this conclusion it is not therefore possible to use such a system to examine whether the presence of host cells influences the response to therapeutic modalities of tumour cells in small spheroids.

I thank Mr. S. Chambers for carrying out the flow cytometric analysis of the cell suspensions.

### References

HAJI-KARIM, M. & CARLSSON, J. (1978). Proliferation and viability in cellular spheroids of human origin. *Cancer Res.*, **38**, 1457.

KRISHAN, A. (1975). Rapid flow cytofluorometric analysis of mammalian cell cycle by propidium iodide staining. *J. Cell Biol.*, **66**, 188.

SIEMANN, D.W., LORD, E.M., KENG, P.C. & WHEELER, K.T. (1981). Cell subpopulations dispersed from solid tumours and separated by centrifugal elutriation. *Br. J. Cancer*, **44**, 100.

STEWART, C.C. & BEETHAM, P.L. (1978). Cytocidal activity and proliferation ability of macrophages infiltrating the EMT6 tumor. *Int. J. Cancer*, **22**, 152.

SUTHERLAND, R.M. & DURAND, R.E. (1976). Radiation response of multicell spheroids: An *in vitro* tumour model. *Curr. Top. Radiat. Res. Q.*, **11**, 87.

TWENTYMAN, P.R. (1980). Response to chemotherapy of EMT6 spheroids as measured by growth delay and cell survival. *Br. J. Cancer*, **42**, 297.

TWENTYMAN, P.R. & WATSON, J.V. (1977). Separation of clonogenic tumour cells from EMT6 mouse mammary tumours. *Br. J. Cancer*, **35**, 120.

VON KLEIST, S., CHANG, E., BARTIN, P., KING, M. & FOGH, J. (1975). Immunohistology of the antigenic pattern of a continuous cell line from a human colon tumour. *J. Natl Cancer Inst.*, **55**, 555.

WARENIUS, H.M. (1980). Identification and separation of mouse and human components of heterotransplanted human tumours. In: *Immunodeficient Animals for Cancer Research.* (Ed. Sparrow), London: Macmillan, p. 207.

YUHAS, J.M., LI, A.P., MARTINEZ, A.O. & LADMAN, A.J. (1977). A simplified method for production and growth of multicellular tumour spheroids. *Cancer Res.*, **37**, 3639.